# *Orostachys japonicus* A. Berger Extracts Induce Immunity-Enhancing Effects on Cyclophosphamide-Treated Immunosuppressed Rats

**DOI:** 10.1155/2019/9461960

**Published:** 2019-01-06

**Authors:** Hak Yong Lee, Young Mi Park, Jeong Kim, Hong Geun Oh, Kang Sung Kim, Hee Joo Kang, Ri Rang Kim, Min Jung Kim, Sang Hee Kim, Hye Jeong Yang, Jisun Oh

**Affiliations:** ^1^INVIVO Co. Ltd., Iksan, Jeollabuk-do 54538, Republic of Korea; ^2^Namwon Herb Agricultural Corp., Namwon, Jeollabuk-do 55770, Republic of Korea; ^3^Huvet Co. Ltd., Iksan, Jeollabuk-do 54630, Republic of Korea; ^4^Department of Food Science and Nutrition, Yong In University, Yongin, Gyeonggi-do 17092, Republic of Korea; ^5^Korea Food Research Institute, Wanju, Jeollabuk-do 55365, Republic of Korea; ^6^School of Food Science and Biotechnology (BK21 Plus), Kyungpook National University, Daegu 41566, Republic of Korea

## Abstract

In this study, we evaluated the immunity-enhancing effects of* Orostachys japonicus* A. Berger (OJ). To examine the immune protective effect in vitro, primary mouse splenocytes were treated with water or ethanol extracts of OJ in the absence or presence of cyclophosphamide (CY), which is a cytotoxic, immunosuppressive agent. The extracts increased the propagation of splenocytes and inhibited CY-induced cytotoxicity. Further, to examine the immunostimulatory effects* in vivo*, adult Wistar rats were orally administered OJ extracts with or without CY treatment. With the administration of OJ extracts, CY-treated immunosuppressed rats showed improved physical endurance, as assessed by the forced swim test. In addition, extract administration increased not only the number of immunity-related cells but also the levels of plasma cytokines. OJ extracts also recovered splenic histology in CY-treated rats. These findings suggest that an OJ regimen can enhance immunity by increasing immune cell propagation and specific plasma cytokine levels.

## 1. Introduction

Cyclophosphamide (CY) is known to possess antitumor and immunomodulatory properties [1, 2]. CY is a cytotoxic agent that disrupts DNA replication and inhibits cell propagation [3, 4] and thus widely used as a chemotherapeutic drug for various types of cancer [2, 5]. In addition, CY can suppress an immune response by modulating lymphocytes [5–8]. Since CY-induced immunosuppression increases vulnerability to pathogen infections and incidence of morbidity and mortality [9–11], adjuvant alternatives in alleviating immunotoxicity and enhancing host immunity are of great interest in cancer therapy [12]. For this reason, the immune-modulating properties of natural plants and their components have been explored for the development of functional foods [9, 13, 14].

*Orostachys japonicus* A. Berger (OJ), known as rock pine (or “Wa-song” in Korean), is a perennial herb belonging to the family Crassulaceae [15] that contains various bioactive compounds such as flavonoids, triterpenoid, and gallic acid [16–22]. OJ is used as a folk remedy, with antioxidant [22–25], anti-inflammatory, and anticancer effects [26–31]. Recently, the immunostimulatory activity of OJ water extract has been studied in RAW264.7 murine macrophage cell line [20]. However, data to support its physiological function are still required.

In this study, we evaluated the immunity-enhancing effect of OJ water and ethanol extracts* in vitro* and* in vivo* using isolated splenocytes and immunosuppressed rats. In particular, the immunomodulatory effects of OJ extracts* in vivo* were assessed by measuring the number of immunity-related cells and the levels of plasma cytokines with or without OJ extract treatment in CY-treated rats.

## 2. Materials and Methods

### 2.1. Preparation of O. japonicus Extracts

Dried OJ (harvested in September 2013; sun-dried) was obtained from the Namwon County Office, Jeollabuk-do, S. Korea. After grinding the dried OJ, including its leaves, stems, and flowers, 100 g of OJ powder was suspended in 1 L of distilled water or 70% ethanol. OJ was extracted from the water or ethanol suspensions by stirring and indirect heating at 100°C or 80°C, respectively, for 3 h. After centrifugation (Beckman, Sanford, ME, USA), at 8000* g* for 30 min each supernatant was filtered through a filter paper (8-*μ*m pore size; Whatman, Little Chalfont, UK), vacuum-evaporated (rotary evaporator RV 10 control, IKA®; Baden-Württemberg, Germany), and freeze-dried (lyophilizer; Il Shin, Seoul, S. Korea) for use in later experiments.

### 2.2. Cell Culture

To obtain splenocytes, spleens were aseptically dissected from 8-week-old Balb/c mice. The tissues were teased apart gently with forceps and then forced through a 70-*μ*m cell strainer (SPL Life Sciences, Pocheon-si, Gyeonggi-do, S. Korea). The resulting cell suspension was washed three times in RPMI-1640 (Invitrogen, Carlsbad, CA, USA) by centrifugation (1,000 g, 5 min, 4°C) and treated with red blood cell lysis buffer (Sigma-Aldrich, St. Louis, MO, USA). The isolated splenocytes were maintained in RPMI-1640-based culture medium supplemented with 10% fetal bovine serum (FBS) and 1% penicillin-streptomycin (all from Invitrogen) in a culture incubator (37°C, 5% CO_2_, humidified).

### 2.3. Cell Viability Assay

To test cell viability after administration of the OJ extracts, Cell Counting Kit-8 (CCK-8; Dojindo Laboratories, Kumamoto, Japan) was used as described previously [32]. Briefly, the cultured splenocytes were harvested, suspended in maintenance medium, and dispensed into a 96-well plate at a density of 2 × 10^5^ in 100 *μ*L per well. After the cells were incubated for 24 h, various concentrations of each extract were applied to the cells in the absence or presence of 1.6 mg/mL of CY (Sigma-Aldrich). After 48 h of cultivation, the CCK-8 assay was performed as instructed by the manufacturer. The absorbance (*Abs*), which is proportional to the number of living cells in each well, was measured at 450 nm using a microplate reader (Infinite 200 PRO, Tecan Group Ltd., Männedorf, Switzerland). Cell viability was calculated with the following equation: Cell viability (%) = (*Abs*_sample_–*Abs*_blank_)/(*Abs*_control_–*Abs*_blank_) × 100.

### 2.4. Experimental Animals

All animal studies were conducted according to the guidelines of the Committee on Care and Use of Laboratory Animals of the Wonkwang University (approval number: WKU15-99). Five-week-old male Wistar rats weighing 120–130 g were purchased from Samtako Inc. (Osan-si, Gyunggi-do, S. Korea). After adaptation for 1 week to a 12-h light/12-h dark regimen (temperature, 23 ± 1°C; humidity, 50 ± 5%; illumination, 150–300 lux), with access to food and water* ad libitum*, 6-week-old rats (150 ± 4 g) were used in this study.

### 2.5. Sample Treatment

Sixty Wistar rats were randomly assigned to six groups (10 rats per group): Untreated (normal), CY-treated (without administration of OJ extract), OJ water extract (OJWE)-low (OJWE administered at 100 mg/kg body weight (BW) with CY treatment), OJWE-high (OJWE administered at 1,000 mg/kg BW with CY treatment), OJ ethanol extract (OJEE)-low (OJEE administered at 100 mg/kg BW with CY treatment), or OJEE-high (OJEE administered at 1,000 mg/kg BW with CY treatment). The OJ extracts were orally administered for 4 weeks using a gastric sonde attached to a syringe on a daily basis. CY was prepared in normal saline and was administered concomitantly with the OJ extracts. To determine the optimal concentration of CY for use in treatments [33], a preliminary* in vivo* experimental study was performed using three different concentrations of CY (5 mg/kg BW, 10 mg/kg BW, and 20 mg/kg BW) in comparison with a control (0 mg/kg BW). Five milligrams of CY/kg BW was determined to be optimal concentration to reduce immunity with minimal effects on body and organ weights and hematology [33, 34] (data not shown).

### 2.6. Forced Swim Test

To assess physiological changes, the forced swim test was performed as described previously [35]. The test was conducted 1 h after the final sample treatment in a Plexiglass cylinder. Prior to the swimming challenge, each animal was weighed, and a weight of about 10% of the animal's BW was placed on its tail. Swimming duration was measured from the time the animal entered the apparatus to 10 sec after the animal stopped moving.

### 2.7. Hematological Assay and Enzyme-Linked Immunosorbent Assays (ELISA)

After the final administration of OJ extracts, the animals were anesthetized with diethyl ether. Whole blood was collected through the abdominal vena cava in ethylenediaminetetraacetic acid (EDTA) microtubes. White blood cell counts for lymphocytes, monocytes/macrophage, and granulocytes (including neutrophils, eosinophils, and basophils) were analyzed using a Hemavet 950 counter (Drew Scientific Group, Dallas, TX, USA). Plasma cytokines, TNF-*α*, IFN-*γ*, and IL-2, were quantified by ELISA kits (R&D Systems, Minneapolis, MN, USA).

### 2.8. Histological Analysis

After each animal was sacrificed, and the organs (liver, kidney, thymus, and spleen) were removed, weighed, and then fixed in 10% neutral buffered formalin. Fixed organs were processed, embedded in paraffin, and cut into 4–7 *μ*m-thick sections using a microtome (Thermo Scientific, Waltham, MA, USA). The sectioned tissues were then stained with hematoxylin and eosin. Tissue damage was observed under an optical microscope (Olympus, Fukuoka, Japan).

### 2.9. Statistical Analysis

The data were analyzed by one-way analysis of variance (ANOVA) and Duncan's multiple range test (SAS software; SAS Institute Inc., USA).* P*-values less than an *α* of 0.05 were considered significantly different. Statistical differences are indicated by letters.

## 3. Results

### 3.1. OJ Extracts Stimulate Splenocyte Proliferation and Protect Splenocytes from CY-Induced Damage

**Figure 1:**
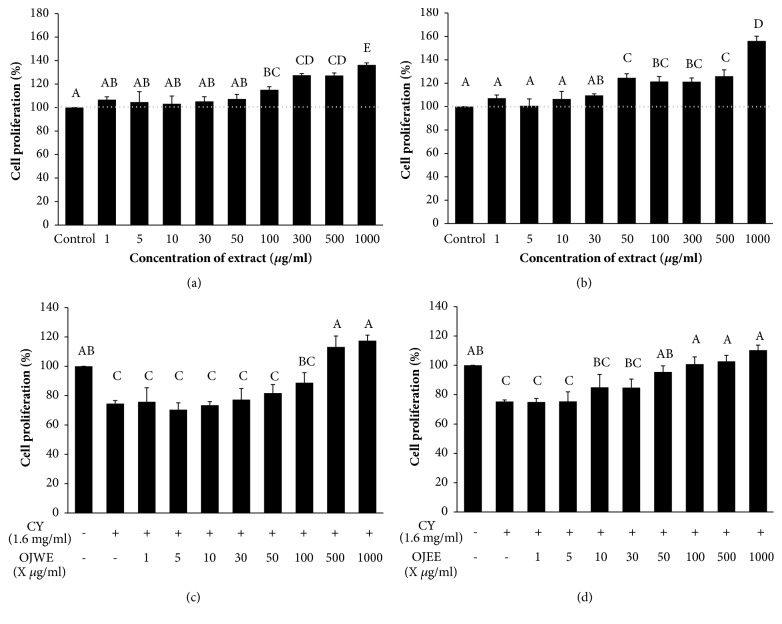
**OJ extracts increased splenocyte proliferation and protected against CY-induced splenocyte damage**. (a–b) The number of isolated splenocytes was augmented with OJWE (a) or OJEE (b) treatment. (c–d) CY-induced splenocyte cytotoxicity was abolished by OJWE (c) or OJEE (d) treatment. Values represent mean ± standard error of the mean (SEM) from three independent experimental sessions (N = 3). Bars not sharing common letter represent statistically significant difference from each other (*p* < 0.05).

To examine the cytotoxicity and cell proliferating effects of both OJWE and OJEE, the isolated splenocytes were treated with various concentrations of the OJ extracts (0, 1, 5, 10, 30, 50, 100, 300, 500, and 1000 *μ*g/mL) for 48 h followed by cell viability testing (Figure 1(a) for OJWE and Figure 1(b) for OJEE). The portion of splenocytes viable increased in a dose-dependent fashion with OJ extract treatment. At 1000 *μ*g/mL of OJWE or OJEE, cell viability was 136.3 ± 1.8% or 156.2 ± 4.1%, respectively, compared to the control (0 *μ*g/mL of OJ extract).

To further examine the cytoprotective effect of OJ extracts against CY-induced damage [4], splenocytes were cultured with the OJ extracts in the absence or presence of CY (Figure 1(c) for OJWE and Figure 1(d) for OJEE). The percent of splenocytes that were viable decreased to about 75% with CY treatment, compared to the control (no treatment). However, cell viability was recovered to the level of the control with treatments of ≥ 500 *μ*g/mL of OJWE or ≥ 50 *μ*g/mL of OJEE.

### 3.2. OJ Extracts Protect the Thymus and Spleen from Weight Loss Resulting from CY Treatment

To evaluate the effects of a 4-week regimen of OJ extract feeding* in vivo*, 6-week-old Wistar rats were orally administered two different concentrations (100 mg/kg BW or 1,000 mg/kg BW) of OJWE or OJEE. Weekly changes in body weight, water intake, and food intake were monitored for each animal and compared among the groups (Supplementary Figure S1A). There were no significant differences observed among the six groups in any of these measures.

After the animals were sacrificed, the weights of the liver, kidney, thymus, and spleen of each animal were measured and then averaged across the group (Supplementary Figure S1B–E). No significant differences in kidney weight were observed among the groups. The liver weight in the group that received CY and/or a high-dose of OJEE was slightly higher than that of the other groups. Interestingly, the weights of the immune-related organs (the thymus and spleen) were similar to those of the control group when OJEE was administered.

### 3.3. OJ Extracts Increase the Swimming Duration and White Blood Cell Counts in Immunosuppressed Rats

**Figure 2:**
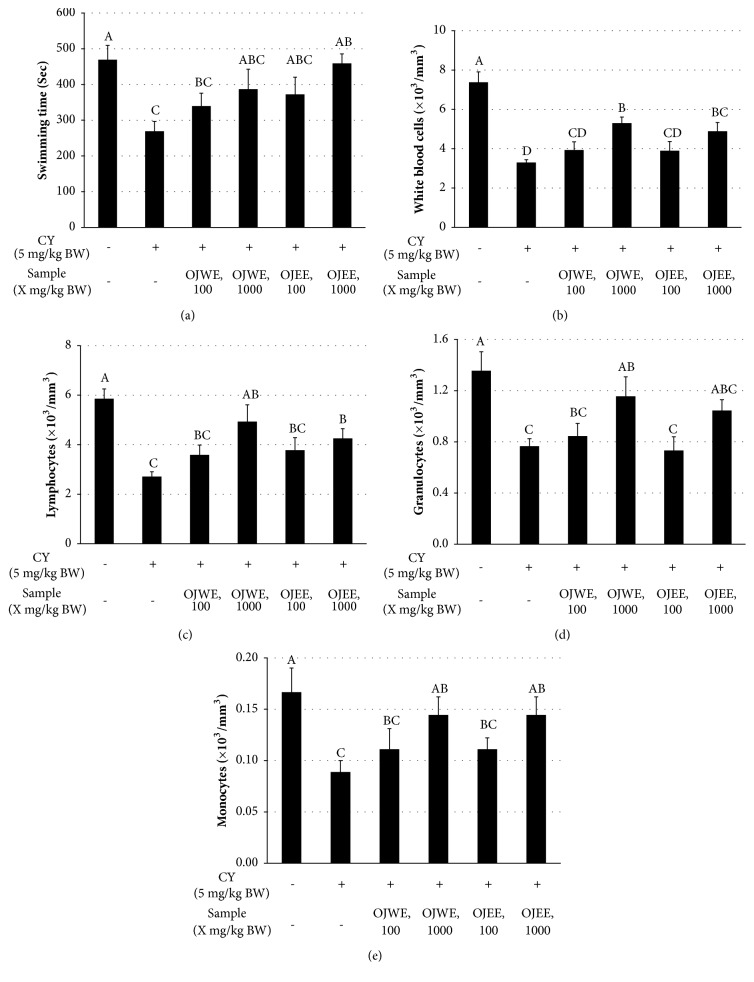
**OJ extracts increased white blood cell counts in immunosuppressed rats**. (a) Swimming duration in the forced swim test. (b–e) White blood cell (WBC) counts: (b) total number of WBCs, (c) lymphocyte counts, (d) granulocyte counts, and (e) monocyte counts. The data obtained from individual animal samples per group were averaged (n = 8); values represent mean ± standard deviation (SD). Bars not sharing common letter represent statistically significant difference from each other (*p* < 0.05).

To evaluate the effect of OJ extracts on resistance against physical stress, the forced swim test was performed. After being fed OJ extracts without or with CY for 4 weeks, rats were allowed to swim, and then the swimming duration of each rat was measured and averaged across the group (Figure 2(a)). We observed that CY treatment decreased the swimming time, while OJ extract treatment tended to increase the time. In particular, a high-dose of OJEE significantly increased the time.

At the end of the OJ extract regimen, a number of WBCs in the blood collected from the sacrificed animals was determined (Figure 2(b)). Total WBC counts were reduced by CY treatment. However, OJ extract treatment caused the counts to rebound in a dose-dependent manner. This phenomenon was observed for the numbers of lymphocytes, granulocytes, and monocytes as well (Figures 2(c)–2(e)).

### 3.4. OJ Extracts Increase the Plasma Levels of Immune-Related Cytokines in Immunosuppressed Rats

**Figure 3:**
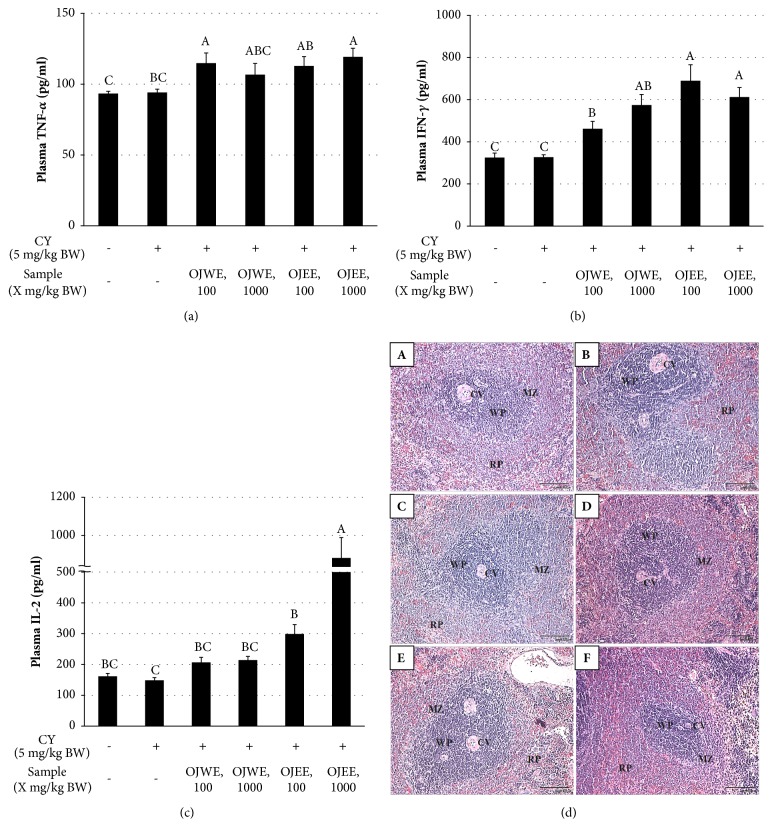
**OJ extracts increased immunity-associated plasma cytokine levels.** (a) Plasma TNF-*α* level. (b) Plasma IFN-*γ* level. (c) Plasma IL-2 level. Values represent mean ± SD (n = 8). Bars not sharing common letter represent statistically significant difference from each other (*p* < 0.05). (d) Representative images of sectioned spleens: (A) normal (no treatment), (B) only CY-treated, (C) OJWE-low, OJWE administered at 100 mg/kg with CY treatment; (D) OJWE-high, OJWE administered at 1,000 mg/kg with CY treatment; (E) OJEE-low, OJEE administered at 100 mg/kg with CY treatment; and (F) OJEE-high, OJEE administered at 1,000 mg/kg with CY treatment. Scale bar in A, 100 *μ*m, applicable to B–F. CV, central vein; MZ, marginal zone; RP, red pulp; WP, white pulp.

To test* in vivo* the effect of OJ extracts on the levels of plasma cytokines related to immunity, the concentrations of TNF-*α*, IFN-*γ*, and IL-2 were measured in the blood collected from animals in all experimental groups (Figures 3(a)–3(c)). The levels of these cytokines in the CY-treated animals were not significantly different from those of the control (no CY treatment). However, the cytokine levels in the OJ extract-fed groups were all higher than those of the groups that were not fed OJ extracts. More specifically, the IFN-*γ* and IL-2 levels were significantly higher in the groups fed OJEE than in those fed OJWE.

Furthermore, spleens were sectioned and histologically analyzed (Figure 3(d)). We found in the normal tissue (i) the white pulp surrounded the central vein and (ii) the marginal zone was observed in between the white pulp and red pulp. In the tissues from the CY-treated group, the marginal zone was obscure, and irregular cell condensates were observed in the red pulp area. However, in the tissues from the groups fed OJ extracts, especially those fed a high concentration of extracts, a clear marginal zone between the white and red pulp was present, and cell condensates were rarely observed. This result indicates that administration of OJ extracts ameliorated CY-induced spleen damage.

## 4. Discussion

In this study, we investigated the activity of OJ water and ethanol extracts on immune enhancement. Both OJ water and ethanol extracts facilitated splenocyte proliferation in a concentration-dependent manner, and the extracts protected splenocytes from CY-induced cytotoxicity. Administration of OJ extracts to CY-treated immunosuppressed rats increased physical endurance, as assessed by the forced swim test. Furthermore, extract administration increased not only WBC counts but also the immunity-related plasma cytokines, TNF-*α*, IFN-*γ*, and IL-2 and recovered normal splenic histology.

CY is an alkylating cytotoxic agent that disrupts DNA replication by crosslinking DNA strands, inhibiting cell propagation and inducing apoptosis [3, 4]. CY is metabolized by cytochrome P450 enzymes, which results in the production of 4-hydroxycyclophosphamide that decomposes into the toxic compound phosphoramide mustard [36, 37]. CY is also known to suppress immune responses by modulating lymphocytes [6, 7]. Thus, CY and its metabolites are commonly used as antineoplastic and immunosuppressive drugs [38]. To generate the immunosuppressed animal model in the present study, we used CY to treat isolated splenocytes as well as Wistar rats.* In vitro* results indicated that OJ extracts could recover splenocyte viability after CY treatment.* In vivo *results showed that administration of OJ extracts could at least in part restore immunity affected by CY treatment, suggesting immune recuperation as a result of extract administration.

There have been many reports of immune disturbances associated with behavioral symptoms in the context of depression [39–41]. The CY-immunosuppressed rats may have been more vulnerable to swim stress than the normal rats [42]. Our data from the blood test showed that treatment with CY, an immunosuppressive agent, reduced the number of WBCs, including lymphocytes, granulocytes, and monocytes/macrophages. However, the reduced WBC counts significantly increased in the high-dose OJ extract-administered individuals. In addition, the duration of forced swimming was reduced by CY treatment but increased in the high-dose OJEE-fed groups. These results suggest that OJ extracts, especially a high-dose of OJEE, may impart resilience against stress by stimulating innate and adaptive immunity.

Consistent with the results that suggest an immunostimulatory function of OJ extracts, the plasma levels of the immunity-related cytokines TNF-*α*, IFN-*γ*, and IL-2 were augmented in CY-treated animals following the administration of OJ extract. In particular, OJEE significantly increased those cytokines compared to OJWE. Cytokines are generally produced by various immune cells and modulate immune responses, such as immune cell survival and differentiation, inflammation, and host defense against bacterial infection [43–45]. In particular, TNF-*α* regulates inflammation and host defense in response to bacterial infection [46] and is suppressed by acute stress [47]. INF-*γ* and IL-2 are produced primarily by T-helper cells [48]. INF-*γ* is a mediator of innate immunity that activates monocytes/macrophages and upregulates major histocompatibility complex (MHC) molecules [49]. IL-2 is a major growth factor for T cells [50] that enhances T cell responses by regulating homeostasis and differentiation of T-regulatory lymphocytes [51]. Thus, the blood test results support the hypothesis that administration of OJ extracts may stimulate innate and adaptive immunity by facilitating the production of immunity-related cytokines.

According to the existing literature, OJ extracts that showed biologically beneficial effects* in vitro* and* in vivo* exerted strong antioxidant activity and contained a plenty of total phenolics and flavonoids [20–22, 25, 26]. Consistently, both OJWE and OJEE were highly capable of scavenging free radicals (data not shown) and rich in flavonoids (Supplementary Table S1). In particular, OJWE and OJEE highly contained gallic acid, catechins, and glycosides of kaempferol and quercetin. However, identification of bioactive components in the extracts that are responsible for immune enhancement awaits further study.

Taken together, our findings from* in vitro* and* in vivo* studies suggest that OJ extracts, especially in ethanol, have immunity-enhancing and host defense effects by increasing the number of immune-related cells and specific cytokines following immunosuppression.

## Data Availability

The datasets generated during and/or analyzed during the current study are available from the corresponding author upon reasonable request.

## Additional Points

*Practical Application. *The activity of water and ethanol extracts of* Orostachys japonicus* A. Berger was investigated on immune enhancement. Our findings indicate that both water and ethanol extracts of* Orostachys japonicus* A. Berger have immunity-enhancing and host defense effects by increasing the number of immune-related cells and specific cytokines following immunosuppression. It would have implications for development of nutraceuticals or functional foods which could aid immunologically vulnerable individuals.

## Supplementary Materials

Supplementary Materials
** Supplementary Figure S1: OJ extracts increased the weights of the thymus and spleen in immunosuppressed animals**. (A) Weekly changes in the body weights, water intake, and food intake of Wistar rats treated or untreated with OJ extracts. Normal, no treatment; Control, only CY-treated; OJWE-low, OJWE administered at 100 mg/kg with CY treatment; OJWE-high, OJWE administered at 1,000 mg/kg with CY treatment; OJEE-low, OJEE administered at 100 mg/kg with CY treatment; OJEE-high, OJEE administered at 1,000 mg/kg with CY treatment. (B–E) Organ weights of OJ extract-treated rats after termination of the 4-week regimen: (B) percent liver weight over BW, (C) percent kidney weight over BW, (D) percent thymus weight over BW, and (E) percent spleen weight over BW. Values represent mean ± SD (n = 8). Bars not sharing common letter represent statistically significant difference from each other (*p* < 0.05).** Supplementary Table S1**: flavonoid contents in the power or extracts of* O. japonica*.
