# Supplementary material for: Orostachys japonicus A. Berger Extracts Induce Immunity-Enhancing Effects on Cyclophosphamide-Treated Immunosuppressed Rats
Source: Biomed Res Int. 2019 Jan 6;2019:9461960. doi: 10.1155/2019/9461960 (PMC6339714; doi:10.1155/2019/9461960)

Supplementary Table 1. Flavonoid contents in the power or extracts of *O. japonica*

Flavonoid*	*O. japonica* powder	*O. japonica* water extract	*O. japonica* ethanol extract
Gallic acid	172.97 ± 20.15	963.96 ± 110.21	335.41 ± 96.51
Apigenin	ND†	ND	ND
Naringenin	0.14 ± 0.03	ND	0.84 ± 0.07
Luteolin	0.60 ± 0.06	0.06 ± 0.01	2.14 ± 0.25
Kaempferol	63.51 ± 7.74	18.65 ± 1.13	168.47 ± 37.90
Epicatechin	64.47 ± 1.78	405.41 ± 17.20	120.72 ± 22.50
Catechin	2.41 ± 0.47	1.38 ± 0.34	4.44 ± 0.03
Quercetin	39.73 ± 1.91	15.14 ± 0.51	114.41 ± 20.89
Chlorogenic acid	0.12 ± 0.00	0.12 ± 0.08	0.15 ± 0.07
Naringin	0.32 ± 0.13	0.39 ± 0.07	0.40 ± 0.05
Rutin	8.32 ± 0.31	16.76 ± 1.27	28.11 ± 2.87
Hesperidin	0.44 ± 0.00	0.44 ± 0.09	0.61 ± 0.03
Protocatechuic acid	13.15 ± 0.61	38.02 ± 4.91	34.77 ± 8.15
Caffeic acid	21.98 ± 1.51	15.32 ± 5.29	25.86 ± 12.93
Methyl caffeate	0.05 ± 0.00	0.03 ± 0.00	0.02 ± 0.00
Hesperetin	ND	ND	0.02 ± 0.00
ECG	477.77 ± 5.10	71.71 ± 3.66	635.14 ± 133.15
Kaempferol-3-glucoside	148.65 ± 9.46	16.40 ± 0.63	330.63 ± 89.50
EGCG	757.66 ± 164.99	16.04 ± 0.62	1441.44 ± 561.14
Quercetin-3-glucoside	520.72 ± 61.47	46.76 ± 0.38	1162.16 ± 346.40

*, Flavonoid contents are presented in units of μg/g; †, ND, not detectable

Supplementary Figure 1

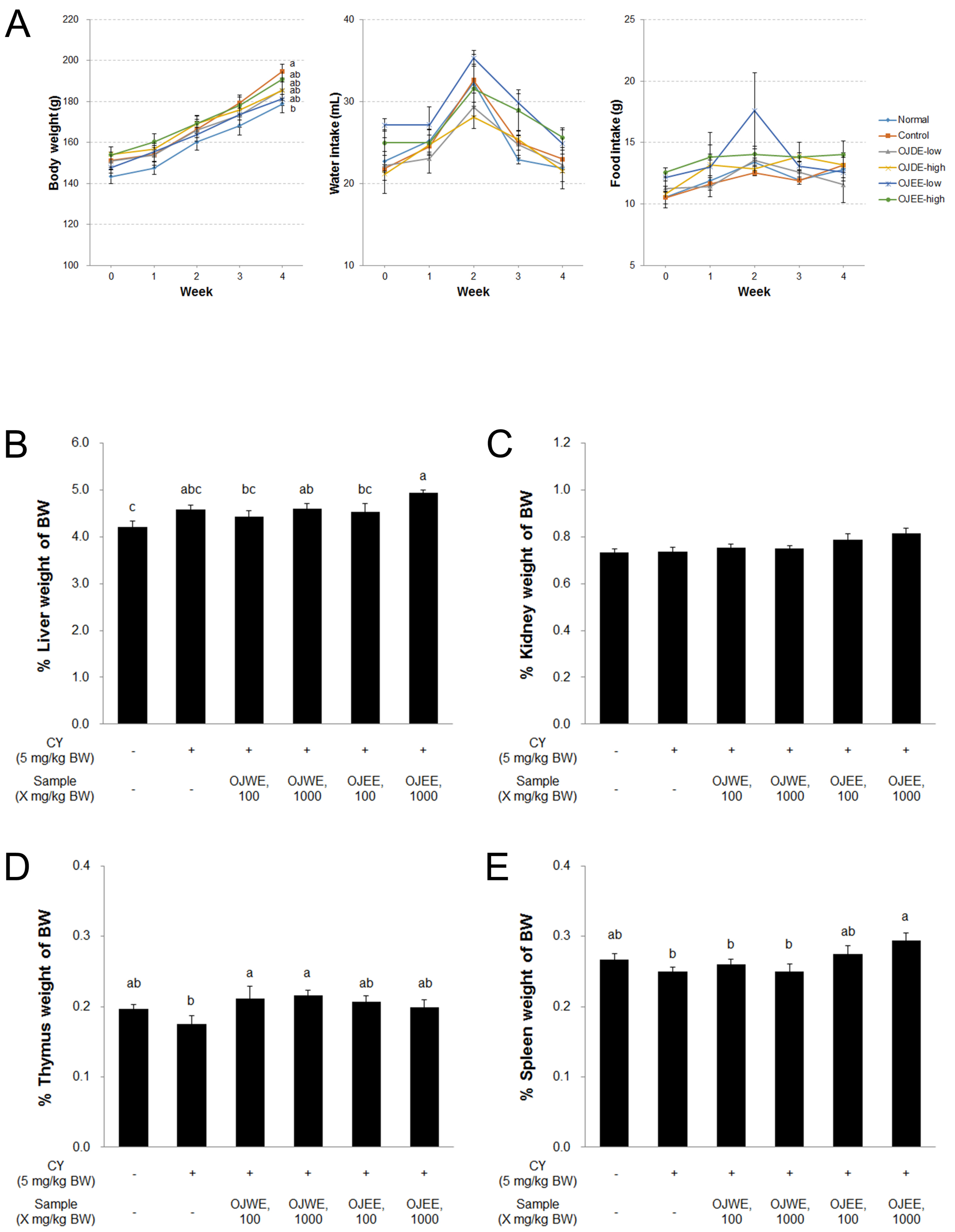

Supplement: Supplementary Materials — Supplementary Figure S1: OJ extracts increased the weights of the thymus and spleen in immunosuppressed animals. (A) Weekly changes in the body weights, water intake, and food intake of Wistar rats treated or untreated with OJ extracts. Normal, no treatment; Control, only CY-treated; OJWE-low, OJWE administered at 100 mg/kg with CY treatment; OJWE-high, OJWE administered at 1,000 mg/kg with CY treatment; OJEE-low, OJEE administered at 100 mg/kg with CY treatment; OJEE-high, OJEE administered at 1,000 mg/kg with CY treatment. (B–E) Organ weights of OJ extract-treated rats after termination of the 4-week regimen: (B) percent liver weight over BW, (C) percent kidney weight over BW, (D) percent thymus weight over BW, and (E) percent spleen weight over BW. Values represent mean ± SD (n = 8). Bars not sharing common letter represent statistically significant difference from each other (p < 0.05). Supplementary Table S1: flavonoid contents in the power or extracts of O. japonica. [file 9461960.f1.pdf]
